# Analysis of OCT Scanning Parameters in AMD and RVO

**DOI:** 10.3390/diagnostics14050516

**Published:** 2024-02-29

**Authors:** Claus von der Burchard, Johann Roider, Timo Kepp

**Affiliations:** 1 Department of Ophthalmology, Kiel University, 24105 Kiel, Germany; johann.roider@uksh.de; 2 German Research Center for Artificial Intelligence, 23562 Lübeck, Germany; timo.kepp@dfki.de

**Keywords:** OCT scanning parameters, biomarker detection sensitivity, field of view, interscan distance

## Abstract

Optical coherence tomography (OCT) is an extensively used imaging tool for disease monitoring in both age-related macular degeneration (AMD) and retinal vein occlusion (RVO). However, there is limited literature on minimum requirements of OCT settings for reliable biomarker detection. This study systematically investigates both the influence of scan size and interscan distance (ISD) on disease activity detection. We analyzed 80 OCT volumes of AMD patients and 12 OCT volumes of RVO patients for the presence of subretinal fluid (SRF), intraretinal fluid (IRF), and pigment epithelium detachment (PED). All volume scans had a scan size of 6 × 6 mm and an ISD of 125 µm. We analyzed both general fluid distribution and how biomarker detection sensitivity decreases when reducing scan size or density. We found that in AMD patients, all fluids were nearly normally distributed, with most occurrences in the foveal center and concentric decrease towards the periphery. When reducing the scan size to 3 × 3 and 2 × 2 mm, disease activity detection was still high (0.98 and 0.96). Increasing ISD only slightly can already compromise biomarker detection sensitivity (0.9 for 250 µm ISD against 125 µm ISD).

## 1. Introduction

Optical coherence tomography (OCT) is the de facto standard for disease monitoring in the most common retinal diseases, including wet age-related macular degeneration (AMD) [1], diabetic macular edema (DME) [2] and retinal vein occlusion (RVO) [3]. For these three disease entities, the first-line therapy is intravitreal injections of medical substrates, which can allow for effective disease control, but needs regular monitoring to determine retreatment necessity or treatment interval.

For these diseases, different biomarkers that can be found in OCT have become surrogate markers for disease activity. Most prominent in current treatment guidelines [4,5,6,7] are central retinal thickness, intraretinal fluid and subretinal fluid (IRF and SRF) in all three diseases and pigment epithelium detachment (PED), which mainly occurs in AMD. Despite the existence of numerous other biomarkers, such as hyperreflective foci, disorganization of inner retinal layers [8], photoreceptor atrophy [9,10], retinal volume [11], and outer retinal tubulations [12], among others, they are not as widely incorporated into clinical practice and are not routinely utilized in disease monitoring. Therefore, most guidelines rely primarily on IRF, SRF, and PED detection, even though modern guidelines and expert consensuses advise to not automatically reject any presence of fluid (tolerating fluids instead of “zero tolerance of fluid”). Rather, the quantity of these fluids should be weighed for a more carefully considered decision [13,14,15,16].

However, even though fluid detection and potentially quantification are the basis of disease monitoring, there is no consensus on exact imaging protocols. While the prevalent OCT systems of big vendors are all equipped with the capability for high-detail imaging, different OCT devices have different standard settings. Today’s common devices exhibit varying scanning densities in their standard modes. For example, Zeiss Cirrus and Topcon DRI OCT Triton typically cover a 6 × 6 mm volume with an interscan distance (ISD) of 47 µm, sampling very densely. In contrast, devices like Heidelberg Spectralis usually scan less densely, but instead repeat the single B-scans for higher quality. This results in a 6 × 6 mm volume and a standard ISD of 125 to 250 µm. Usually, the scanning parameters can further be individually modified by the end user, leading to even more diversity among different clinical practices. Since typically, follow-up examinations will be performed with the same device and settings, clinical interpretability is commonly given within the same practice, but interoperability between different physicians often is limited. 

Various scan sizes and densities obviously have a significant influence on the sensitivity of biomarker detection. In cases of small field of view (FOV) or low density (i.e., high ISD), biomarkers present only in small quantities or off-centered might be overlooked. There is lacking knowledge of how these parameters influence disease detection sensitivity and which parameters would be considered sufficient or optimal for disease monitoring.

Therefore, we analyzed datasets of AMD and RVO patients with high imaging standards and systematically examined how reducing imaging settings would influence the biomarker detection rate.

## 2. Materials and Methods

### 2.1. Datasets

As the basis for OCT requirement analysis in AMD patients, we used the high-quality OCT reference volumes of 68 eyes of 40 AMD patients from a study investigating a home care OCT device [17]. Within this study, all patients with the diagnosis of an exudative AMD under current anti-VEGF treatment were included, regardless of which type of CNV was present. All of these images were taken with a Heidelberg Spectralis OCT scanner (Heidelberg Engineering, Heidelberg, Germany) with a FOV of 6 × 6 mm with 49 B-scans, corresponding to 125 µm ISD. All scans were annotated for the presence of SRF, IRF, and PED, as well as a segmentation of the total retina from ILM to Bruch’s Membrane, by JF (see Acknowledgements) and TK as junior graders and by CB as senior grader.

**Figure 1:**
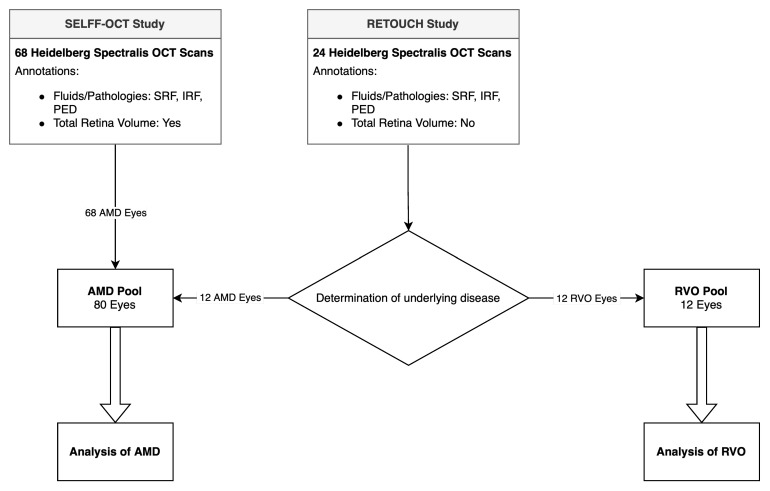
Flowchart of data acquisition and processing.

We enlarged this dataset by inclusion of the published training dataset of the RETOUCH challenge [18]. The RETOUCH study provides annotated OCT images of AMD and RVO patients taken with different OCT devices. For reasons of comparability, we only included the dataset taken by the Heidelberg Spectralis OCT. These images were acquired with the same imaging settings as the first dataset. All of the RETOUCH images had already been annotated for fluids by at least one expert (for details, see [18]). The annotation settings were also comparable to our annotations so that both datasets could be used interchangeably. Since for the RETOUCH dataset, no information was provided or could be obtained from the study’s authors regarding which scan had which underlying disease, all scans were graded for the underlying disease (AMD or RVO) based on the presence of biomarkers and clinical expertise by CB. In the end, 12 scans of AMD patients and 12 scans of RVO patients could be included. Figure 1 illustrates data acquisition and processing.

Because of the matching imaging settings, the AMD datasets were combined for an overall analysis of 80 OCT volumes in AMD patients. Because of the small sample size of only 12 RVO patients, the analysis mainly focused on AMD patients. For RVO, the data are discussed in the manuscript, but the figures visualizing the data are moved to the Supplementary Data section in order to maintain good readability of the manuscript. 

The RETOUCH dataset provided only annotations for IRF, SRF, and PED, not for the total retina. For analysis of total retinal volume, therefore, analysis could only be performed for the subset of 68 AMD subjects.

For both data sets, ethics approval was obtained before data acquisition and analysis [17,18].

### 2.2. Image Preprocessing

All data preprocessing was performed automatically via a custom Python script. For all analysis, the actual OCT image data were ignored, and only the segmentation files were processed, since these contained all necessary information. All left eyes were mirrored horizontally, so that the nasal portion was always to the right and the temporal portion to the left.

### 2.3. Data Analysis

The data analysis was also performed via custom Python scripts. All data analysis was performed separately for the biomarkers IRF, SRF, and PED. Since many treatment guidelines consider any presence of IRF and SRF equally, we also performed pooled analysis for IRF and SRF combined (IRF ∪ SRF). 

First, we created heatmaps to understand the distribution of different types of fluid. For each fluid, this process involved first summing up the individual scans of each eye along the z-axis (en face view). This resulted in one 2D biomarker heatmap per eye. Second, this 2D heatmap was binarized so that it accounted only for the presence of a given fluid at one location, without consideration of fluid height or quantity. Then, all of these binarized segmentation files were summed up to represent a biomarker heatmap for all patients. This sum was divided by the overall number of patients, so that the resulting heatmap showed a fraction from 0 to 1, indicating how many eyes showed the presence of a biomarker at a given point. For better interpretation, an early treatment diabetic retinopathy study (ETDRS) grid was projected on the resulting visualizations.

Following the initial findings indicating a Gaussian normal distribution, the heatmaps were then compared by a curve fit to a perfect Gaussian distribution and an R^2^ value was calculated. For this, the following Gaussian function was defined:

(1) $$f\left(x,y\right)=A\cdot \exp\left(-\frac{{\left(x-\mu_{x}\right)}^{2}+{\left(y-\mu_{y}\right)}^{2}}{2\sigma^{2}}\right),$$

where A corresponds to the amplitude, $\mu_{x}$ and $\mu_{y}$ to the mean position, and σ to the standard deviation. Then, the best curve fit of this function to the results and the corresponding R^2^ value were calculated. 

Second, we analyzed how different FOVs would influence the rate of biomarker detection. For this purpose, we systematically cropped the volumes in 250 µm segments independently on the x- and y-axis. Then, the sensitivity for the tested biomarker across all volumes was compared to the whole volume. The results are depicted on a sensitivity map, where for each coordinate, the sensitivity in comparison to the 6 × 6 mm volume is shown color coded. The sensitivities for a 3 × 3, 2 × 2, and 1 × 1 mm volume are shown and highlighted as exemplary reduced FOVs.

Third, we analyzed how changing ISD would influence sensitivity and volume measurements. For this, incrementally every second B-scan was dropped, and sensitivity was determined against the original scan. To calculate the volume, the number of 2D voxels of a certain biomarker in each scan was simply multiplied by the ISD as a simple approximation of the total 3D volume. To account for the potential impact of border effects, the first and last B-scan that lay at the border of the volume were only multiplied with half the ISD. This ensured that the overall analyzed volume would stay constant regardless of ISD. 

To quantify the difference in biomarker volume from scans with an increased ISD in comparison to the original scan, the mean absolute percentage error (MAPE) was calculated as follows:

(2) $$\mathrm{MAPE}=\frac{1}{n}\sum_{t=1}^{n}\left|\frac{Reference_{t}-Reduced\_ISD_{t}}{Reference_{t}}\right|$$

## 3. Results

### 3.1. Frequency of Biomarkers in Data Collection

Of the 80 AMD scans, IRF was present in 20 scans (25%), SRF was present in 44 (55%), SRF or IRF in 48 scans (60%) and PED was present in 72 scans (90%). For the 12 RVO scans, IRF was present in 12 scans (100%), SRF in 5 scans (42%), SRF or IRF in 12 scans (100%) and PED in 0 scans (0%).

### 3.2. Biomarker Distribution

**Figure 2:**
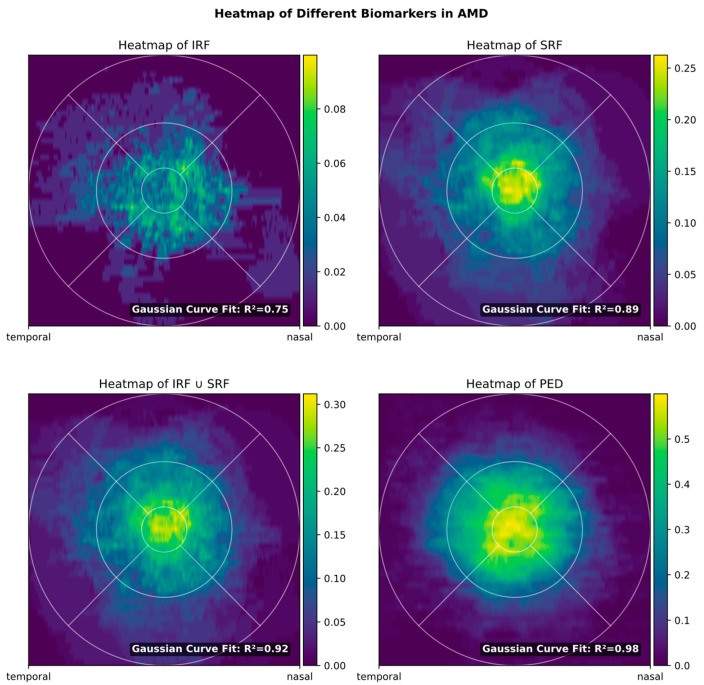
Heatmap of biomarkers for AMD in the en face view. The color scale, ranging from 0 to 1, illustrates the proportion of images where a specific biomarker was identified at the corresponding location. Notably, different color bars are employed for different biomarkers, reflecting variations in biomarker prevalence among the images.

The heatmap shows that in AMD, all biomarkers were symmetrically distributed, with peak prevalence in the foveal region (Figure 2). Whereas PED and SRF showed an almost perfect Gaussian distribution (R^2^ = 0.89 for SRF and 0.98 for PED), this pattern was less pronounced for IRF (R^2^ = 0.75), where the prevalence within the central 3 mm ETDRS grid was nearly identical. This might be explained by the foveal architecture with less pronounced inner retinal layers (where IRF accumulation is mostly found) in the center. 

For RVO, the results were similar (Supplementary Figure S1). It could be shown that IRF was much more frequent. Similarly to the AMD findings, also in RVO patients, SRF was more normally distributed (R^2^ = 0.86) than IRF (R^2^ = 0.68). PED, in contrast, did not occur in our RVO dataset.

### 3.3. Influence of FOV on Biomarker Detection

**Figure 3:**
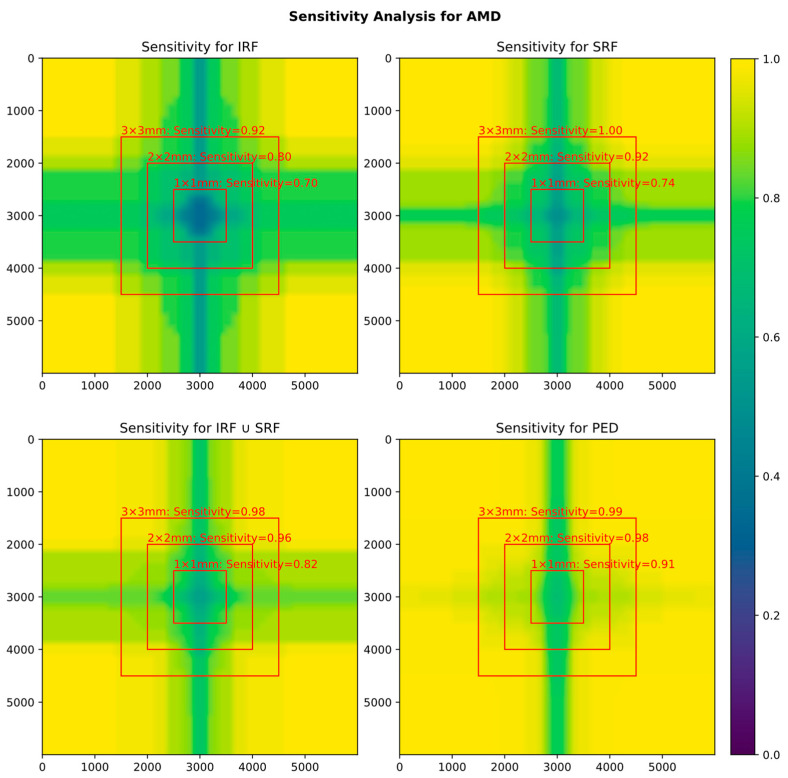
Influence of scan size (FOV) on biomarker detection. Yellow = sensitivity 1.0; blue: sensitivity 0. The red squares show exemplary sensitivities for a scan size of 3 × 3, 2 × 2 and 1 × 1 mm.

As could be expected, reducing the FOV incrementally also reduces biomarker detection sensitivity. However, in good accordance with the biomarker heatmap (Figure 2) that shows most occurrences in the center, most loss in sensitivity only occurs when reducing the field of view further than 3 × 3 mm (Figure 3).

In an injection scheme where treatment necessity was indicated by the presence of IRF or SRF, the sensitivity of detecting either fluid would be 0.98 for a 3 × 3 mm grid and still 0.96 for a 2 × 2 mm grid. 

Again, the results were similar for RVO, where the detection rate of IRF or SRF even stayed at 1.0 for both a 3 × 3 and a 2 × 2 mm fovea-centered scan (Supplementary Figure S2). 

### 3.4. Influence of Interscan Distance (ISD)

**Figure 4:**
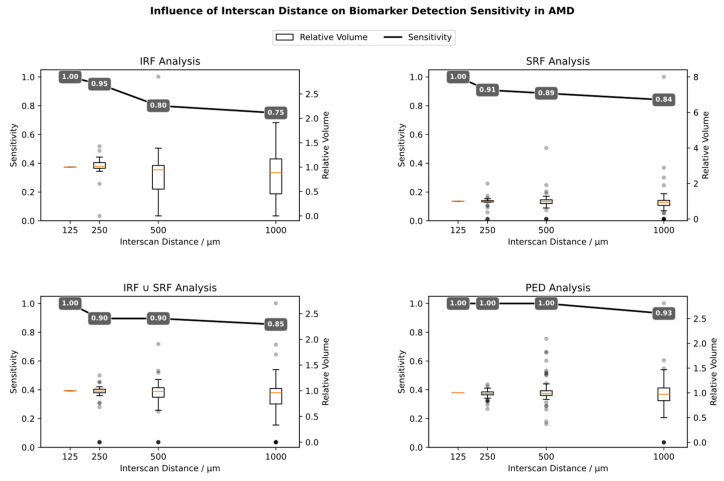
Relationship of Interscan Distance to Sensitivity and Relative Volume Measurements for AMD. The black line shows the sensitivity of detecting the biomarker in question when the ISD is increased. The boxplot shows how the single biomarker volume measurements diverge when ISD is increased (red line: median value, grey dots: individual outliers).

When analyzing sensitivity for biomarker detection when ISD is increased, it can be shown that the sensitivity for both SRF and IRF detection already drops with the first increment from 125 to 250 µm (Figure 4). Moreover, the individual biomarker volume measurements already vary drastically even with the first increment, highlighting that biomarker volume quantifications of scan settings with different ISDs are not comparable. Table 1 quantifies the influence of ISD on Mean Absolute Percentage Error (MAPE) for each biomarker volume.

For RVO, increasing ISD seems to be less compromising both for biomarker detection sensitivity and volume measurement. The biomarker detection sensitivity stays at 1.0 for both IRF and SRF when increasing the ISD to 250, 500 and even 1000 µm (Supplementary Figure S3), and also the MAPE for biomarker volume quantification is less affected (Table 1). n/a: Data not available.

### 3.5. Influence of ISD on Total Retinal Volume

**Figure 5:**
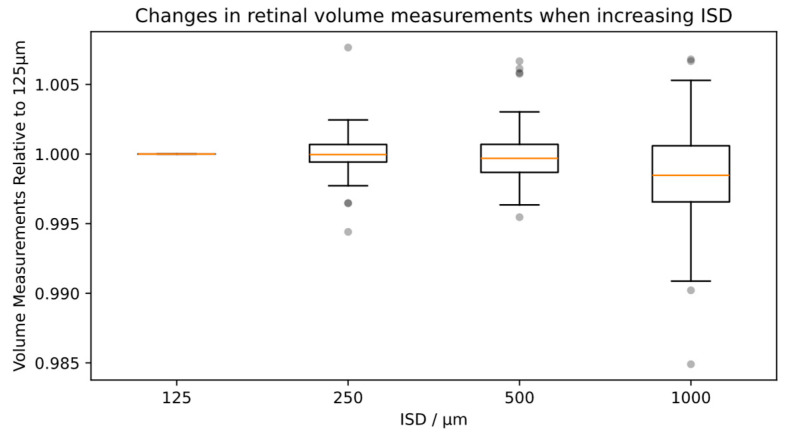
Relative volume measurements for total retinal volume when increasing ISD (red line: median value, grey dots: individual outliers).

When measuring total retinal volume, it can be shown that the total retinal volume measures do not change significantly when increasing ISD (Figure 5). Compared to the original volume with 125 µm ISD, the mean absolute percentage difference is 0.1% for 250 µm, 0.15% for 500 µm and 0.32% for 1000 µm (see Table 1, last column).

## 4. Discussion

### 4.1. Motivation

Macular OCT scans are the most common imaging modality in modern ophthalmology. Considering the widespread, high-frequency application of OCT, surprisingly little data exist on minimum requirements. As our data show, reducing scan size or scan density will compromise biomarker detection sensitivity and volumetric measurements. At the same time, there are several reasons to not unnecessarily inflate imaging protocol:

- From a patient’s perspective, the acquisition time will increase. Especially in cases of poor fixation because of advanced macular disease, longer acquisition time can quickly become bothersome, especially since the examination potentially must be repeated every other month.
- From the device operator’s perspective, the increased acquisition time is also bothersome and reduces efficiency and productivity. Additionally, not only acquisition but also processing and storage time are prolonged.
- From the ophthalmologist’s perspective, more image material means longer loading time and more data to interpret.
- Also, due to poor fixation or tear film breakup, image quality might shrink with longer acquisition time, so that the advantages of high-resolution widefield OCT might be outweighed by worse image quality.

All of this suggests that reasonably low scan density and scan size settings with high image quality might be better than a densely scanned, large volume scan of low to medium quality images. The more abundant the number of single B-scans, the higher also the chance that the ophthalmologist will overlook abnormalities. Artificial intelligence (AI)-based approaches can offer a solution to this challenge by effectively highlighting scans where specific biomarkers are detected. This automated assistance can enhance the accuracy and efficiency of biomarker identification [19,20]. In fact, especially for effective training of neural networks, it is crucial to have large datasets of densely sampled OCT scans with a high FOV. Even for end-user applications, neural networks trained on high-quality scans stand to gain from elevated quality standards. Therefore, the potential future applications of AI should be carefully balanced against the motivation to reduce scanning parameters.

### 4.2. Implications for Home Monitoring OCT Devices

New monitoring paradigms such as home care OCT might come with different scanning parameters, which should be properly evaluated. Reducing the scanning settings can simplify the OCT design and, therefore, be beneficial for both cost reduction and device miniaturization. Notal Vision has proposed an OCT home monitoring solution with a scan size of 3 × 3 mm and an ISD of 34 µm [21], i.e., a very dense, but small size OCT scan. According to our data, this reduced FOV will still allow for detection of 98% of SRF ∪ IRF, which per se sounds very reasonable. In a cross-sectional study of 469 successful self-scans [22], the authors identified only three cases (0.6%) where in the reference 6 × 6 mm scan, fluid was found exclusively outside the central 3 × 3 mm area. This means that 99.4% of fluid would still be detectable with the reduced FOV, closely mirroring our prediction. Regarding individual biomarkers, the authors did not present distribution details in the reference OCT but focused solely on detection rates using the investigational device. While this approach can provide a reasonable estimate, it may be affected by false negatives and positives. For IRF, the authors found a detection sensitivity of 91%, which almost exactly matches our predicted value of 92%. For SRF, the paper had a slightly lower sensitivity of 93% instead of the predicted 100%. However, as mentioned above, this lower sensitivity could also be due to image quality or other factors that cannot be extracted from the published data. Overall, the data of this large cross-sectional analysis align remarkably well with our data. 

Another home monitoring OCT approach co-developed by our group is SELFF-OCT [17], which provides very high scan density (8 µm), but an even smaller field of view of 3×1.8 mm. According to our analysis, this would allow for a combined detection rate for SRF ∪ IRF of 96%, which again closely resembles the actual study’s findings of a sensitivity of 92%. However, while according to the data of this manuscript, the SRF detection rate should be expected to be reasonably high (93%), the IRF detection rate would only be found at 85%, which might be considered deficient. In this case, the actual SRF detection rate of 90% almost mirrors the prediction, whereas IRF detection was distinctly lower at only 57%. This discrepancy is most likely explained by other factors than FOV reducing sensitivity, mainly image quality. 

Overall, the results of these two studies align with our analysis, supporting the suggestion that smaller FOVs than 6 × 6 mm are likely acceptable for home-monitoring purposes. Still, the potential benefit in cost or size reduction should very carefully be weighed against the reduction in sensitivity and deviation from the de facto clinical standard of 6 × 6 mm. Outside of special imaging circumstances such as home monitoring OCT, we do not recommend reducing the FOV.

### 4.3. Comparison to Similar Studies

The influence of ISD on biomarker detection sensitivity has been analyzed before. Fang et al. [23] reviewed a mixed dataset of AMD, RVO and DME patients with a very dense scanning protocol (ISD 60 µm) and found almost no difference when reducing to 120 µm ISD (sensitivity still 99%). When increasing scan distance to 240 or even 480 µm, sensitivity still stayed at 97% and 87%, respectively. This is slightly more favorable towards increasing ISD than our AMD data (which found a sensitivity of 90% for both 250 and 500 µm ISD, see Figure 2). However, it must be considered that patients with RVO and DME were also included in said study. Our data shows that RVO is less sensitive to increasing ISD; clinical experience suggests comparable effects for DME, even though we cannot provide conclusive data. This might explain the smaller effect for increasing ISD in the mentioned study with a mixed patient collective. In another study of 59 AMD patients [24], the authors found detection rates of 91% for SRF and 90% for IRF when increasing the ISD from 47 to 188 µm, which is roughly comparable to our findings. A study in 394 AMD eyes investing a star-shaped radial scan pattern with only 12 B-scans found that this radial pattern shows fluid detection sensitivity similar to that of a 25 B-scan 250 µm ISD raster volume [25]. Interestingly, this study found that both scan patterns missed some fluids that were found in the other modality, which again highlights that 250 µm ISD will overlook fluid in AMD. As for total retinal volume, multiple prior studies have found that it can be measured reliably with high ISDs of around 500 µm or more [26,27,28], which corresponds well to our data.

Regarding the influence of FOV on biomarker detection sensitivity, we found no other study that systematically analyzes this other than a small study also conducted by our group [29]. In this study, we manually rated 293 volume scans of 16 AMD patients. All scans were 6 × 6 mm with 250 µm ISD. In a blinded and randomized order, the uncropped 6 × 6 mm was rated whether fluid was present or not or uncertain. Then, also a cropped 3 × 3, 2 × 2 and 1 × 1 mm volume with the same ISD was rated. When excluding all images rated uncertain, the study found a sensitivity of 97.8% for 3 × 3 mm, 95.7% for 2 × 2 mm and 88.7% for 1 × 1 mm. The findings of this manual analysis are almost identical to the automated analysis presented in this paper.

### 4.4. Authors’ Suggestion for Scanning Parameters

Our data show that even small compromises in ISD lead to reduced detection of relevant biomarkers in AMD, and biomarker volume quantifications quickly become unreliable. Similarly, in RVO, even though the biomarker detection rate is not as quickly affected by ISD, volume measurements also begin to vary with increased ISD. Parallel results for other retinal diseases seem plausible.

In a time where AI assistants for the physician or even AI-based fully automated OCT reports have become increasingly available and important in routine care, we believe that the benefits of a small ISD outweigh the potential concerns discussed in Section 4.1. Therefore, we suggest using an ISD of 125 µm or lower as the routine setting for macular OCT scans.

As for scan size, our analysis shows that a moderate reduction of scan size does not impact disease activity detection rates in AMD and RVO monitoring. However, this does not mean that no relevant information is lost with size reduction. Also, we have shown that the FOV reduction is possible in these two diseases because they are highly concentrated in the foveal and perifoveal region; this will not necessarily hold up for other diseases. Since 6 × 6 mm historically already has been incorporated as a de facto standard by almost all device manufacturers, we suggest keeping this standard in routine use.

### 4.5. Limitations

The sample size in this paper, chiefly for RVO patients, was limited due to unavailability of more annotated data. However, especially for the bigger AMD dataset, we believe to have sufficient data for substantial conclusions, as can be shown by the good accordance with the preexisting literature. Another limitation is that no DME data were available. Moreover, it must be considered that both datasets were acquired in Europe. In other regions, presentation of AMD might differ (e.g., more cases of polypoidal choroidal vasculopathy in Asia [30]), which might limit the transferability to other regions.

It could be hypothesized that even with a relatively dense ISD of 125 µm, small biomarker occurrences might be overlooked. Theoretically, an even denser sampled volume as ground truth would be favorable, but data were not available. However, Fang et al. [23] showed almost no differences between 60 and 120 µm ISD, as discussed above.

We only analyzed OCT data acquired with a Heidelberg Spectralis OCT device. The RETOUCH dataset theoretically also provides data from two other devices (Topcon T-1000/T-2000 and Zeiss Cirrus) which were recorded with a smaller ISD of 47 µm. However, these datasets are comparably small, and since 125 µm is no multiple or close-multiple of 47 µm, proper joint analysis cannot be performed. Given the high and comparable image quality of modern OCT devices, we would not expect any significant differences. Also, cursory analysis showed no hints towards distinctly different findings.

## Figures and Tables

**Table 1:** Effect of ISD on Mean Absolute Percentage Error (MAPE) for Different Biomarker Volumes and Total Retinal Volume in AMD and RVO.

Mean Absolute Percentage Error (MAPE) When Increasing ISD					
ISD (µm)	Biomarker Volume				
	IRF	SRF	IRF or SRF	PED	Total Retina
AMD Patients					
250	15.49%	18.14%	15.58%	5.17%	0.10%
500	42.64%	31.68%	22.52%	14.92%	0.15%
1000	45.72%	53.14%	36.30%	26.42%	0.32%
RVO Patients					
250	9.37%	1.17%	9.07%	n/a	n/a
500	5.42%	4.31%	5.03%	n/a	n/a
1000	25.39%	31.48%	25.53%	n/a	n/a

## Data Availability

Source data are unavailable. For the SELFF-OCT dataset, informed consent of the patients does not allow for publication of the images. For the RETOUCH dataset, data access can be requested from the study’s authors.

## Supplementary Materials

The following supporting information can be downloaded at: https://www.mdpi.com/article/10.3390/diagnostics14050516/s1, Figure S1: Influence of scan size (FOV) on biomarker detection in RVO; Figure S2: Sensitivity analysis for decreased FOV in RVO; Figure S3: Relationship of Interscan Distance on Sensitivity and Relative Volume Measurements for RVO.
