# Supplementary material for: Analysis of OCT Scanning Parameters in AMD and RVO
Source: Diagnostics (Basel). 2024 Feb 29;14(5):516. doi: 10.3390/diagnostics14050516 (PMC10931062; doi:10.3390/diagnostics14050516)
Supplement: Supplementary file 1 [file diagnostics-14-00516-s001.zip › diagnostics-2880605-supplementary.pdf]

Analysis of Fluid Distribution and OCT Disease Activity Detection Requirements in AMD and RVO

Supplementary Material

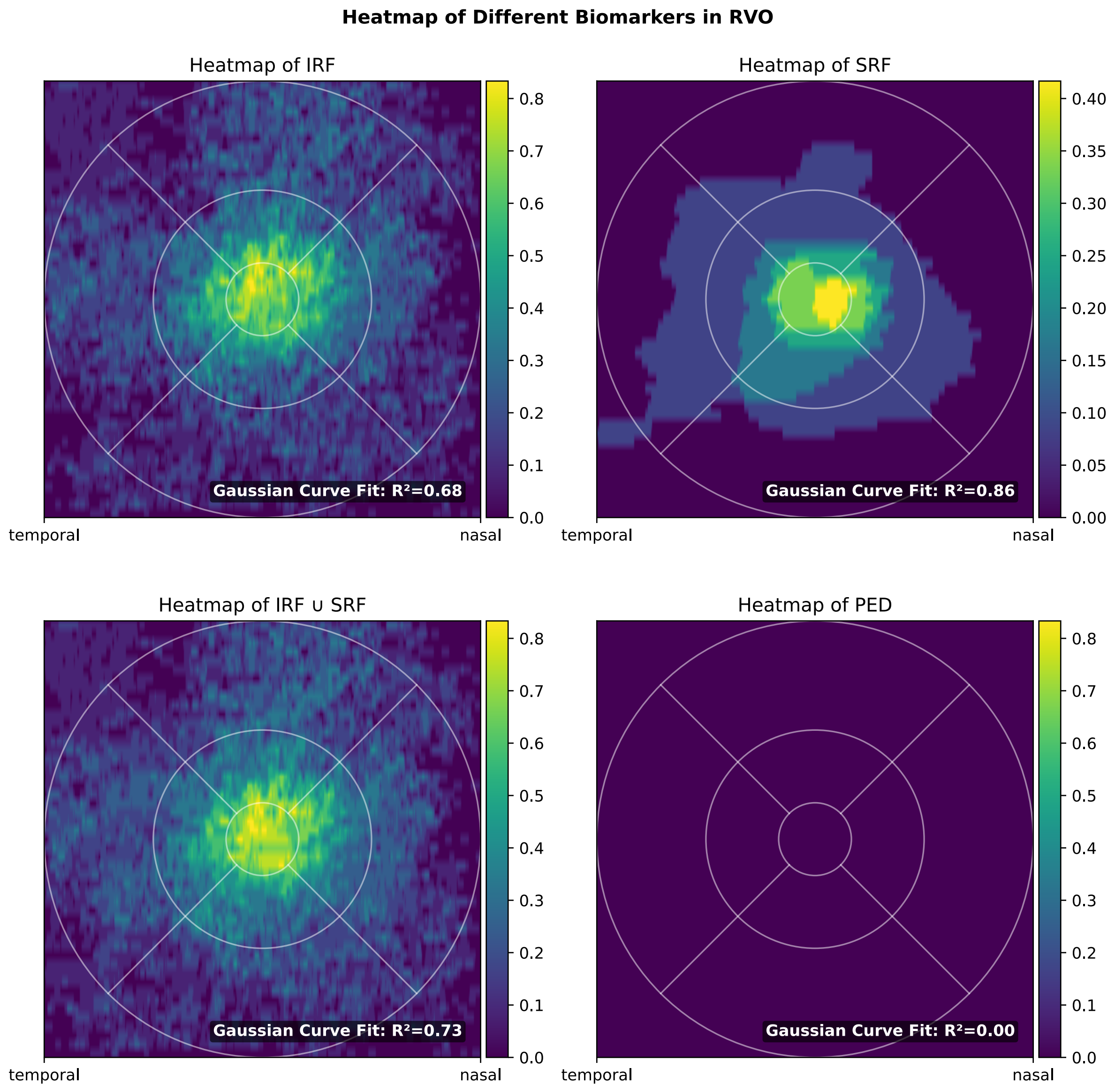

Figure S1. Heatmap of biomarkers in RVO in the en face view (corresponding to Figure 1 for AMD patients). The color scale, ranging from 0 to 1, illustrates the proportion of images where a specific biomarker was identified at the corresponding location. Notably, different color bars are employed for different biomarkers, reflecting variations in biomarker prevalence among the images.

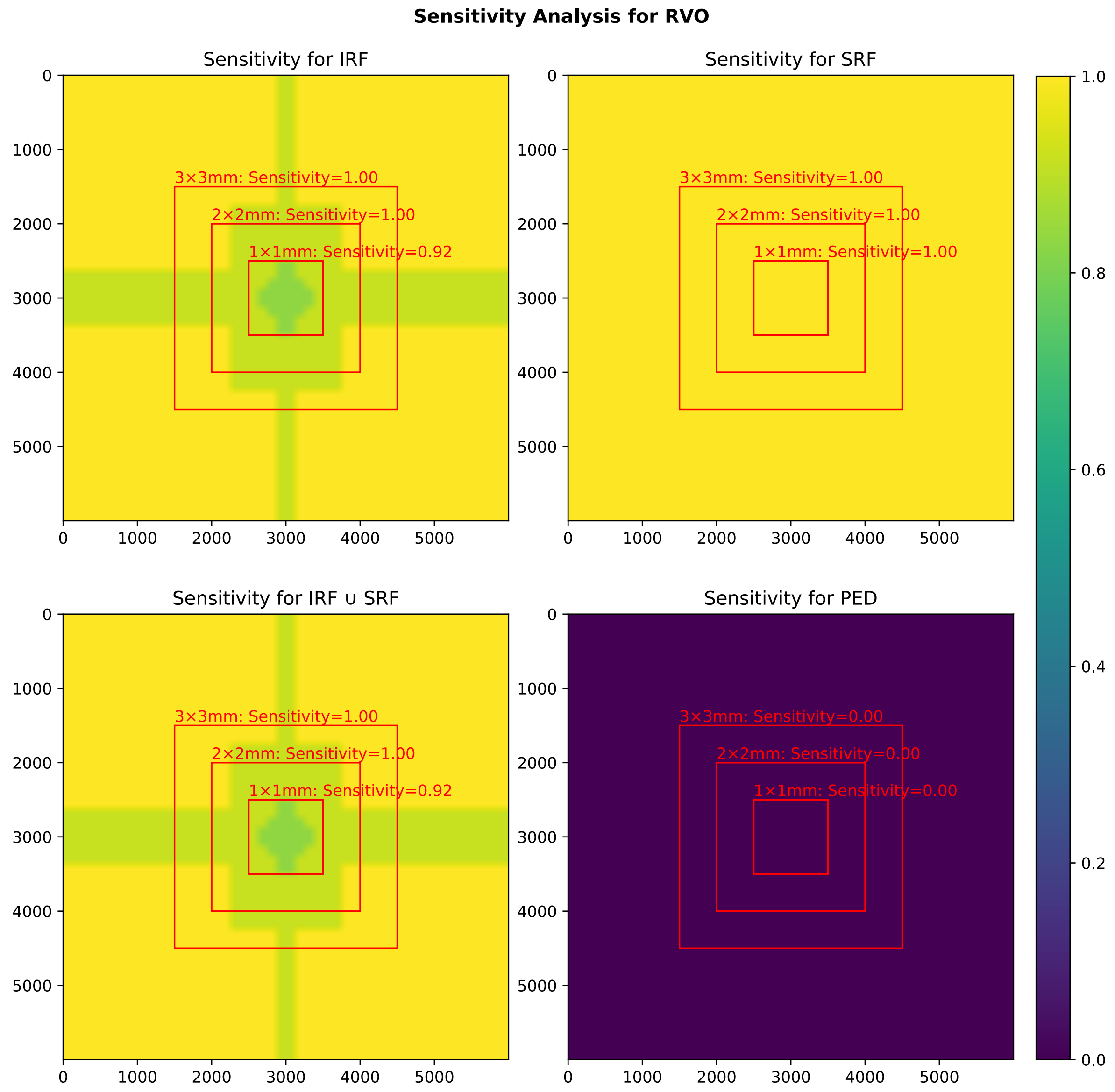

Figure S2. Influence of scan size (FOV) on biomarker detection in RVO (corresponding to Figure 2 in AMD patients). Yellow = sensitivity 1.0; blue: sensitivity 0. The red squares show exemplary sensitivities for a scan size of 3×3, 2×2 and 1×1mm.

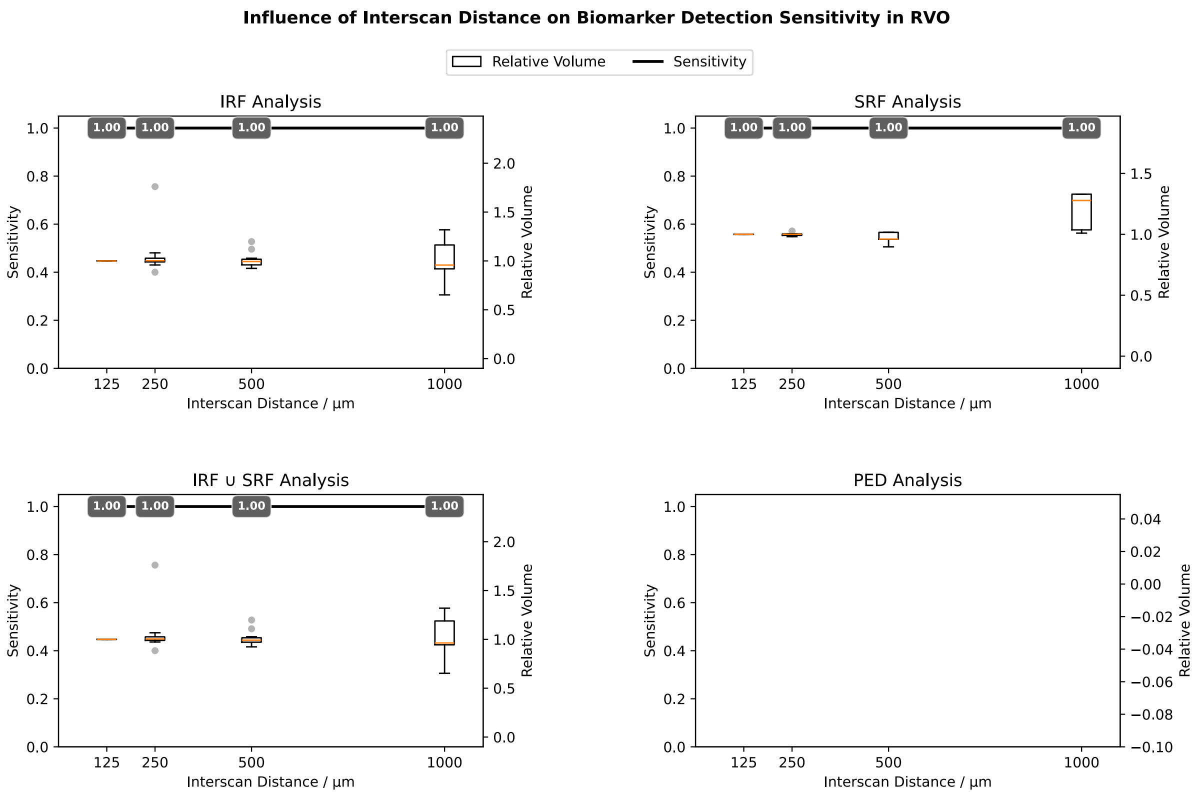

Figure S3. Relationship of Interscan Distance on Sensitivity and Relative Volume Measurements for RVO (corresponding to Figure 3 in AMD patients). The black line shows the sensitivity of detecting the biomarker in question when the ISD is increased. The boxplot shows how the single biomarker volume measurements diverge when ISD is increased (red line: median value, grey dots: individual outliers).
